# Temperature-related mortality impacts under and beyond Paris Agreement climate change scenarios

**DOI:** 10.1007/s10584-018-2274-3

**Published:** 2018-09-13

**Authors:** Ana Maria Vicedo-Cabrera, Yuming Guo, Francesco Sera, Veronika Huber, Carl-Friedrich Schleussner, Dann Mitchell, Shilu Tong, Micheline de Sousa Zanotti Stagliorio Coelho, Paulo Hilario Nascimento Saldiva, Eric Lavigne, Patricia Matus Correa, Nicolas Valdes Ortega, Haidong Kan, Samuel Osorio, Jan Kyselý, Aleš Urban, Jouni J. K. Jaakkola, Niilo R. I. Ryti, Mathilde Pascal, Patrick G. Goodman, Ariana Zeka, Paola Michelozzi, Matteo Scortichini, Masahiro Hashizume, Yasushi Honda, Magali Hurtado-Diaz, Julio Cruz, Xerxes Seposo, Ho Kim, Aurelio Tobias, Carmen Íñiguez, Bertil Forsberg, Daniel Oudin Åström, Martina S. Ragettli, Martin Röösli, Yue Leon Guo, Chang-fu Wu, Antonella Zanobetti, Joel Schwartz, Michelle L. Bell, Tran Ngoc Dang, Dung Do Van, Clare Heaviside, Sotiris Vardoulakis, Shakoor Hajat, Andy Haines, Ben Armstrong, Kristie L. Ebi, Antonio Gasparrini

**Affiliations:** 1 Department of Public Health, Environments and Society, London School of Hygiene & Tropical Medicine, Keppel St, Bloomsbury, London, WC1E 7HT UK; 2 Department of Epidemiology and Preventive Medicine, School of Public Health and Preventive Medicine, Monash University, 99 Commercial Road, Melbourne, VIC 3004 Australia; 3 Division of Epidemiology and Biostatistics, School of Population Health, University of Queensland, St Lucia, Brisbane, QLD 4072 Australia; 4 Potsdam Institute for Climate Impact Research, Telegrafenberg, 14473 Potsdam, Germany; 5 Universidad Pablo de Olavide, Carretera de Utrera, 41013 Sevilla, Spain; 6 Climate Analytics, Ritterstraße 3, 10969 Berlin, Germany; 7 School of Geographical Sciences, University of Bristol, University Road, Bristol, BS8 1SS UK; 8 School of Public Health and Institute of Environment and Human Health, Anhui Medical University, Meishan Road, Hefei, 81 230032 China; 9 Shanghai Children’s Medical Centre, Shanghai Jiao-Tong University, 1678 Dongfang Rd, Shanghai, 200127 China; 10 School of Public Health and Social Work, Queensland University of Technology, 2 George St, Brisbane City, QLD 4000 Australia; 11 Institute of Advanced Studies, University of São Paulo, Rua Praça do Relógio, 109, Building K, 5th floor, Cidade Universitária, ZC, São Paulo, São Paulo 05508-970 Brazil; 12 School of Epidemiology and Public Health, University of Ottawa, 600 Peter Morand Crescent, Ottawa, K1G 5Z3 Canada; 13 Healthy Environments and Consumer Safety Branch, Health Canada, Ottawa, Canada; 14 Department of Public Health, Universidad de los Andes, Mons. Alvaro del Portillo 12, 455 Santiago, Chile; 15 Department of Environmental Health, School of Public Health, Fudan University, 138 Yi xue yuan Road, Shanghai, 200032 China; 16 Department of Environmental Health, University of São Paulo, Av. Dr. Arnaldo, 715 - Cerqueira César, São Paulo, São Paulo 03178-200 Brazil; 17 Institute of Atmospheric Physics, Academy of Sciences of the Czech Republic, Bocni, 1401 14131 Prague, Czech Republic; 18 Faculty of Environmental Sciences, Czech University of Life Sciences, Kamycka, 129 16521 Prague, Czech Republic; 19 Center for Environmental and Respiratory Health Research, University of Oulu, Aapistie 5B, FI-90014 Oulu, Finland; 20 Santé Publique France, French National Public Health Agency, 12 rue du Val d’Osne, 94415 Saint Maurice, France; 21 School of Physics, Dublin Institute of Technology, Kevin Street 2, Dublin, D08 X622 Ireland; 22 Institute of Environment, Health and Societies, Brunel University London, Kingston Ln, Uxbridge, London, UB8 3PH UK; 23 Department of Epidemiology, Lazio Regional Health Service, Via Cristoforo Colombo, 112 00147 Rome, Italy; 24 Department of Pediatric Infectious Diseases, Institute of Tropical Medicine, Nagasaki University, 1-12-4 Sakamoto Nagasaki, Nagasaki, 852-8523 Japan; 25 Faculty of Health and Sport Sciences, University of Tsukuba, 1-1-1 Tennodai, Tsukuba, 305-8574 Japan; 26 Department of Environmental Health, National Institute of Public Health, Universidad No. 655 Colonia Santa María Ahuacatitlán, Cerrada Los Pinos y Caminera, 62100 Cuernavaca, Morelos Mexico; 27 Department of Environmental Engineering, Graduate School of Engineering, Kyoto University, Kyoudai Katsura Campus, Nishikyou Ward, Kyoto, 615-8540 Japan; 28 Department of Global Ecology, Graduate School of Global Environmental Studies, Yoshidahonmachi, Sakyo Ward, Kyoto, 606-8501 Japan; 29 Graduate School of Public Health, Seoul National University, 1Gwanak-ro Gwanak-gu, Seoul, 08826 Republic of Korea; 30 Institute of Environmental Assessment and Water Research (IDAEA), Spanish Council for Scientific Research (CSIC), Jordi Girona 18-26, 08034 Barcelona, Spain; 31 Department of Statistics and Computational Research, Environmental Health Joint Research Unit FISABIO-UV-UJI CIBERESP, University of Valencia, Valencia, Spain; 32 Department of Public Health and Clinical Medicine, Umeå University, 901 85 Umeå, Sweden; 33 Swiss Tropical and Public Health Institute, Socinstrasse 57, 4051 Basel, Switzerland; 34 University of Basel, Petersplatz 1, 4001 Basel, Switzerland; 35 Environmental and Occupational Medicine, and Institute of Occupational Medicine and Industrial Hygiene, National Taiwan University (NTU) and NTU Hospital, 1 Section 4, Roosevelt Rd, Da’an District, Taipei, Taiwan; 36 National Institute of Environmental Health Sciences, National Health Research Institutes, 35 Keyan Road, 35053 Zhunan, Taiwan; 37 Department of Environmental Health, Harvard T.H. Chan School of Public Health, 677 Huntington Ave, Boston, MA 02115 USA; 38 School of Forestry and Environmental Studies, Yale University, 195 Prospect St, New Haven, CT 06511 USA; 39 Faculty of Public Health, University of Medicine and Pharmacy, Ho Chi Minh city, 217 Hồng Bàng, Phường 11, Quận 5, Ho Chi Minh City, Vietnam; 40 Institute of Research and Development, Duy Tan University, 254 Nguyễn Văn Linh, Thạc Gián, Q. Thanh Khê, Da Nang, Vietnam; 41 Chemical and Environmental Effects, Centre for Radiation, Chemical & Environmental Hazards, Public Health England, Didcot Oxon, Chilton, London, OX11 0RQ UK; 42 Institute of Occupational Medicine, Research Avenue North, Riccarton, Edinburgh, EH14 4AP UK; 43 Center for Health and the Global Environment (CHanGE), University of Washington, Seattle, WA 98105 USA

**Keywords:** Climate change, Mortality, Temperature, Projections

## Abstract

The Paris Agreement binds all nations to undertake ambitious efforts to combat climate change, with the commitment to “hold warming well below 2 °C in global mean temperature (GMT), relative to pre-industrial levels, and to pursue efforts to limit warming to 1.5 °C”. The 1.5 °C limit constitutes an ambitious goal for which greater evidence on its benefits for health would help guide policy and potentially increase the motivation for action. Here we contribute to this gap with an assessment on the potential health benefits, in terms of reductions in temperature-related mortality, derived from the compliance to the agreed temperature targets, compared to more extreme warming scenarios. We performed a multi-region analysis in 451 locations in 23 countries with different climate zones, and evaluated changes in heat and cold-related mortality under scenarios consistent with the Paris Agreement targets (1.5 and 2 °C) and more extreme GMT increases (3 and 4 °C), and under the assumption of no changes in demographic distribution and vulnerability. Our results suggest that limiting warming below 2 °C could prevent large increases in temperature-related mortality in most regions worldwide. The comparison between 1.5 and 2 °C is more complex and characterized by higher uncertainty, with geographical differences that indicate potential benefits limited to areas located in warmer climates, where direct climate change impacts will be more discernible.

**Electronic supplementary material:**

The online version of this article (10.1007/s10584-018-2274-3) contains supplementary material, which is available to authorized users.

## Background

The Paris Agreement, adopted in December 2015 under the auspices of the United Nations Framework Convention on Climate Change (UNFCCC), is a turning point in negotiations to limit the potential damages of climate change. An agreement was reached on an ambitious long-term goal to limit warming from pre-industrial levels “well below” a 2-°C increment in global mean temperature (GMT), and to pursue efforts to limit it to 1.5 °C (UNFCCC 2015a; UNFCCC 2015b). The UNFCCC invited the Intergovernmental Panel on Climate Change (IPCC) to provide a special report in 2018 on the impacts of 1.5- vs. 2-°C warming, to determine the avoided impacts of further reductions in greenhouse gas emissions. This report will include multisectoral assessments of the regional-scale consequences for society and environment of different degrees of warming, with comprehensive evaluations of potential impacts. Non-optimal outdoor temperatures will be included in the assessment of impacts on human health (Seneviratne et al. 2016). Most studies projecting the health risks of temperature extremes were performed in country-specific settings, mainly in high-income regions (Sanderson et al. 2017). The majority of these studies focused on heat-related deaths, and did not consider cold-related mortality, which may be reduced as temperature continues to increase (Wang et al. 2016). Our recent multi-country health impact projection analysis, the largest of its kind, investigated trends in temperature-related mortality under various greenhouse gas (GHG) emission trajectories (Gasparrini et al. 2017). However, these studies offered little evidence on the comparative risks under warming scenarios based on specific GMT targets, such as those under the Paris Agreement. The present contribution addresses this research gap by assessing the excess mortality attributable to non-optimal temperature projected across various geographical and climate regions under climate change scenarios corresponding to 1.5 and 2 °C increases in GMT, and by comparing them with more extreme warming scenarios corresponding to 3 and 4 °C increases.

## Brief description of the method

We projected temperature-related excess mortality in 451 locations from 23 countries across the globe characterized by a wide range of climatic conditions, included in the Multi-Country Multi-city (MCC) Collaborative Research Network (http://mccstudy.lshtm.ac.uk/) (Table 1, Fig. S1, and Supplementary Material S1). We applied a methodology developed in a previous study on trends in temperature-mortality projections performed within the same collaborative network (Gasparrini et al. 2017). Here in this section, we provide a brief description of this methodological framework. For a more detailed explanation on the different analytical steps, refer to the Supplementary Material S2 and to a previous work (Gasparrini et al. 2017). In brief, we firstly estimated the location-specific exposure-response relationship between observed daily temperature and mortality counts using historical data collected through the MCC Network. We then projected temperature-related excess mortality, using the modeled daily series of temperature and mortality, under scenarios consistent with 1.5, 2, 3, and 4 °C increases in GMT above pre-industrial levels, assuming no change in demographics or population vulnerability. Thus, results are expressed as function of specific warming levels, instead of calendar periods under specific emission pathways as done in the previous publication (Gasparrini et al. 2017). These warming scenarios were defined using 20-year windows of modeled daily temperature within 1990–2099 corresponding to specific GMT increases. These “time-slices” were obtained from three global climate models (GCMs, specifically HadGEM2-ES (Jones et al. 2011), IPSL-CM5A-LR (Mignot and Bony 2013), and MIROC-ESM-CHEM (Watanabe et al. 2011)) under Representative Concentration Pathway 8.5 (RCP8.5). The combination of RCP8.5 and the three GCMs was selected to ensure a warming level of up to 4 °C in GMT over the current century. The time slices were identified in each GCM following a procedure detailed elsewhere (Schleussner et al. 2016). See Table S2 for the specific periods, Table 1, and Fig. S2 for the geographical distribution of the location-specific GMT increase from 1.5 to 2 °C and Fig. S3 for the overall GCM-ensemble trends. This time-slice approach is justified given that little evidence for scenario dependence was found for temperature-related indices and a warming of up to 4 °C (Seneviratne et al. 2016; James et al. 2017). At the same time, by using location-specific series, we account for differential increases in temperature across geographical areas for each GMT scenario. Location-specific excess mortality, expressed in terms of attributable fractions for cold (below the minimum mortality temperature, MMT) and heat (above MMT), in each 20-year window were averaged across GCMs to obtain the corresponding ensemble estimate in each scenario (Gasparrini and Leone 2014).

**Table 1:** Description of the countries included in the study, listed by geographic region, with the number of locations, average and range of the mean temperatures modeled in the baseline period (1986–2005), the average difference between the projected mean temperatures between 2 and 1.5 **°**C increases in GMT, and classification of the locations in each country per climate zone

Geographic region	Country	Number of locations	Average [range]^a^ daily mean temp. baseline	Difference in average daily mean temp. 2 vs 1.5 °C scen.	*N* [%] locations per climate zone^b^			
					Equatorial	Dry	Warm temperate	Snow
North America	Canada	26	6.6 [10.5; 2.6]	0.87	0 [0%]	0 [0%]	3 [11.5%]	23 [88.5%]
	USA	135	14.9 [25.5; 8.0]	0.74	5 [3.7%]	11 [8.1%]	85 [63%]	34 [25.2%]
Central America	Mexico	10	18.5 [23.0; 13.5]	0.69	0 [0%]	5 [50%]	5 [50%]	0 [0%]
South America	Brazil	18	24.3 [27.2; 17.5]	0.55	14 [77.8%]	0 [0%]	4 [22.2%]	0 [0%]
	Chile	4	13.2 [14.8; 11.3]	0.32	0 [0%]	0 [0%]	4 [100%]	0 [0%]
North Europe	Finland	1	5.7 [5.7; 5.7]	0.72	0 [0%]	0 [0%]	0 [0%]	1 [100%]
	Ireland	6	9.8 [10.6; 9.1]	0.51	0 [0%]	0 [0%]	6 [100%]	0 [0%]
	Sweden	1	7.6 [7.6; 7.6]	0.66	0 [0%]	0 [0%]	0 [0%]	1 [100%]
	UK	10	10.0 [11.3; 9.2]	0.60	0 [0%]	0 [0%]	10 [100%]	0 [0%]
Central Europe	Czech Republic	4	8.5 [9.3; 7.8]	0.63	0 [0%]	0 [0%]	4 [100%]	0 [0%]
	France	18	12.2 [15.9; 10.3]	0.63	0 [0%]	0 [0%]	18 [100%]	0 [0%]
	Moldova	4	10.0 [10.5; 9.5]	0.61	0 [0%]	0 [0%]	3 [75%]	1 [25%]
	Switzerland	8	10.0 [12.5; 8.1]	0.61	0 [0%]	0 [0%]	7 [87.5%]	1 [12.5%]
South Europe	Italy	11	15.2 [18.2; 12.3]	0.60	0 [0%]	0 [0%]	11 [100%]	0 [0%]
	Spain	52	15.3 [21.6; 10.8]	0.69	0 [0%]	7 [13.5%]	45 [86.5%]	0 [0%]
East Asia	China	15	14.9 [23.6; 7.0]	0.75	0 [0%]	2 [13.3%]	8 [53.3%]	5 [33.3%]
	Japan	47	15.3 [23.1; 9.0]	0.72	0 [0%]	0 [0%]	41 [87.2%]	6 [12.8%]
	South Korea	7	13.7 [14.9; 12.4]	0.72	0 [0%]	0 [0%]	5 [71.4%]	2 [28.6%]
South-East Asia	Philippines	4	27.9 [28.5; 27.6]	0.48	4 [100%]	0 [0%]	0 [0%]	0 [0%]
	Taiwan	3	24.0 [25.2; 23.2]	0.50	1 [33.3%]	0 [0%]	2 [66.7%]	0 [0%]
	Thailand	62	27.4 [29.0; 24.9]	0.65	62 [100%]	0 [0%]	0 [0%]	0 [0%]
	Vietnam	2	26.6 [28.0; 25.3]	0.59	2 [100%]	0 [0%]	0 [0%]	0 [0%]
Australia	Australia	3	18.0 [20.2; 15.6]	0.45	0 [0%]	0 [0%]	3 [100%]	0 [0%]

^a^Range corresponds to the maximum and minimum average value of the daily mean temperatures of the baseline period estimated across locations

^b^Climate zone defined according to Köppen-Geiger classification (Kottek et al. 2006), using the first letter: A, equatorial; B, dry; C, warm temperate; D, snow. Any location included in the study was classified as E, polar, so this category was omitted

We estimated differences in temperature-related excess mortality in 2, 3 and 4 °C scenarios relative to 1.5 °C for heat, cold, and total (net, i.e., the summed contribution of heat and cold). Heterogeneity was explored by aggregating absolute and differences in excess mortality for each scenario by country, geographic region, and climate zone (defined by the first letter of the Köppen-Geiger classification (Kottek et al. 2006)).

## Results

**Fig. 1:**
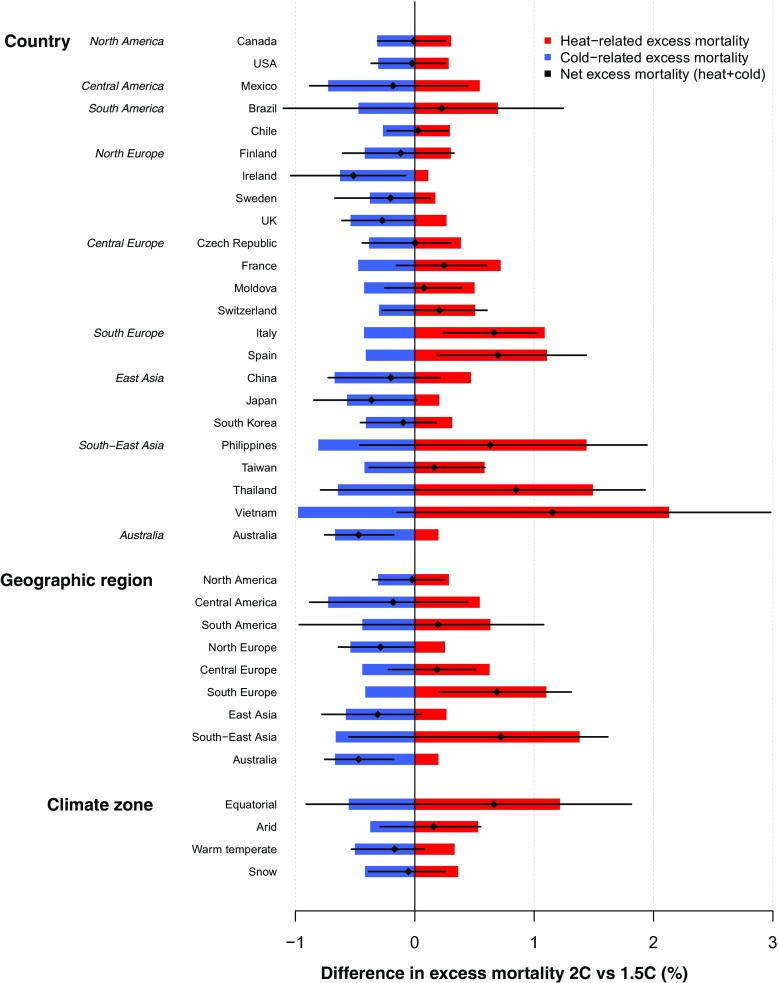
Change in excess mortality from 1.5- to 2-°C scenario by country, geographic region and climate zone. Red and blue bars represent changes in heat (above minimum mortality temperature) and cold (below minimum mortality temperature) excess mortality, respectively, and black diamond and bar correspond to net excess mortality (heat+cold) and 95% confidence interval.

Figure 1 displays the projected changes in excess mortality by comparing the 1.5- vs 2-°C increase scenarios in each country, geographic region, and climate zone (actual numbers in Tables S3–S6). Overall, an increase from 1.5 to 2 °C in GMT could generate a substantial rise in heat-related mortality in most countries included in the analysis. Specifically, assuming no changes in population and vulnerability, heat-mortality impacts could increase between + 0.11 and + 2.13%, with most countries in South Europe and South-East Asia showing increments above + 1%. In contrast, cold-related mortality could decrease in all countries between − 0.27 and − 0.98%. These decrements are of a lower magnitude compared to the corresponding heat-related risks, producing a net increase in excess mortality in about half of the countries but with large uncertainty in most of them. For instance, only Italy and Spain, the two countries in the South of Europe included in the study, could face a significant net increase in total excess temperature-related mortality of about + 0.66 and + 0.77%, respectively. Similar or larger but not statistically significant increases, reaching + 1.15%, could be observed in other warm countries in South-East Asia, such as the Philippines, Thailand, and Vietnam, despite having different climate conditions (Fig. 1, Fig. S1, Table 1). In contrast, total excess mortality could decrease in cooler areas such as North Europe and East Asia. For example, Ireland and Japan could register a small decrease between − 0.51 and − 0.27%. Other large countries (Canada, USA, and Chile), and some countries located in central areas such as Czech Republic and Moldova, might not face substantial changes in total excess mortality. However, many of the net change estimates are not statistically significant, reflecting the uncertainty in the estimates.

**Fig. 2:**
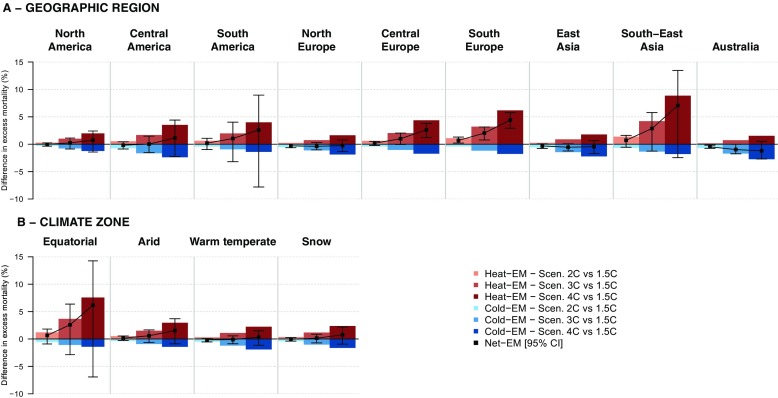
Trends in changes in excess mortality projected for warming in 2, 3 and 4 °C, relative to 1.5 °C, by geographic region and climate zone. Red and blue bars represent changes in heat (above minimum mortality temperature) and cold (below minimum mortality temperature) excess mortality, respectively, while black squares correspond to net excess mortality (heat+cold) and its 95% confidence interval.

Figure 2 illustrates the trends in differences in excess mortality by geographic region and climatic zone projected under other warming scenarios, including those beyond the Paris Agreement targets, corresponding to increases in GMT of 2, 3, and 4 °C, relative to 1.5 °C (figures in Tables S3–S6). As expected, we observed a common pattern of rising mortality associated with heat and a moderate attenuation in cold-related impacts. Under more extreme scenarios, most regions could experience considerably larger heat-mortality risks that would not be balanced by the projected decreases in cold-related excess mortality. Specifically, central and southern regions of America, Europe, and East-Asia are projected to experience increases in heat-related mortality impacts ranging between + 3.53 and + 8.86% in the most extreme 4-°C scenario, while cooler regions in Europe and Asia could face smaller increases below + 2%. In the latter regions, the contribution of the reduction in cold-related mortality impacts would be similar to the increase observed for heat, ranging between − 1.88 and − 2.23%, translating into small or even null and non-significant net reductions in total excess mortality. The most affected areas in terms of large increases in net temperature-mortality impacts would be again the warmest regions in the south of Europe and South-East-Asia (+ 4.41 and + 7.07%), and in a smaller magnitude in South America and Central Europe (+ 2.60 and + 2.63%). However, estimates on net changes are highly uncertain for most of the regions and warming levels, showing statistically significant results only for the central and southern European regions. A different pattern is observed for Australia, with negative net changes in excess mortality across the warming scenarios. This would be due to the milder temperature projections obtained for this region, compared to other areas of similar climate, as shown in Fig. S3 and in a previous work (Gasparrini et al. 2017).

**Fig. 3:**
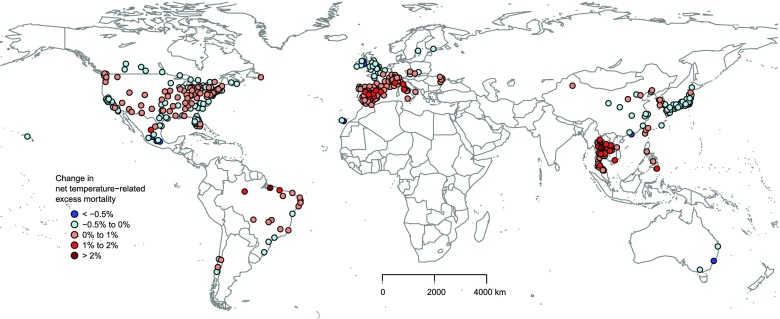
Map showing the geographical distribution of the location-specific total excess mortality change between 1.5 and 2 °C scenarios

The pattern of results suggests an association between projected total temperature impacts and current climate conditions. This is particularly clear in Europe and Asia, where the impact of a change in temperature applied to the current population shows moderate decreases in excess mortality in the colder areas of the north, nearly null changes or small increases in the temperate central areas, and larger increases in warmer southern regions. This pattern is better illustrated in Fig. 3 showing the geographical distribution of the location-specific estimates for the 2- vs 1.5-°C difference. We can also observe a within-country positive gradient from temperate to warmer or equatorial areas in Brazil, although this was not clear in other countries in Central America and USA; the results obtained by different climate zones confirm this gradient (Figs. 1 and 2, bottom panel). Although imprecise, our projections indicate that locations in equatorial climates could experience a + 0.66% increase in total excess mortality from 1.5 to 2 °C warming, followed by a smaller + 0.16% increase in arid zones, while cooler regions (warm temperate and snow climates) could register a decrease or close-to-null change in total excess temperature-related mortality (Fig. 1, Table S6) based on the current population. Similarly, equatorial locations would be the most affected areas by far under more extreme scenarios, experiencing an increment in total mortality impacts above + 6% if GMT increases by 4 °C.

Figure S4 shows the results from a sensitivity analysis in which we compare the GCM-specific and ensemble excess mortality estimated for each geographic area for 1.5-°C warming scenario. We observe little variation across models, thus confirming that impact calculation does not depend on the timing in which each GCM reach a specific warming level, and therefore on the choice of specific climate models or emission pathways.

## Study context and interpretation of the results

This study investigates the potential temperature-mortality impacts projected under specific climate change scenarios consistent with the Paris Agreement. Projections are presented as a function of GMT rise, instead of climate trajectories or emission scenarios as in previously published health impact assessments. Our approach is consistent with the framework of the Paris Agreement, which sets explicit temperature targets but does not define specific timelines or mitigation plans. It is therefore implicit that the same GMT rise can be reached under different development pathways and mitigation policies, as previously demonstrated (Seneviratne et al. 2018). Coherently, impact estimates are likely to be largely independent from the choice of alternative GCM/RCP combinations to select the time slices corresponding to a specific GMT increase, as shown in our analysis (Fig. S4). While this framework offers a straightforward way to quantify target-related impacts, it does not permit the integration of other factors that are likely to modify future health burdens, such as demographic changes and adaptation strategies, as the timing and socio-economic pathways leading to the related GMT increases can vary. By contrast, the applied methodology is based on clear assumptions defining simplified scenarios of stable populations and no-adaptation. Although we acknowledge that these are unrealistic representations of plausible futures, these simplified scenarios facilitate the proper interpretation of the results, as the impact due to global warming can be disentangled from the contribution of other factors. Consistently, our estimates should not be considered predictions of future mortality, but projections of hypothetical health impacts based on current socio-economic conditions, population structure and level of vulnerability. While recognizing these limitations, findings from this study can serve as a basis in the ongoing discussions on the implementation of the Paris Agreement, and can be then extended and refined to assess more specific scenarios that take into account detailed mitigation strategies and underlying changes in baseline populations and temperature-related risks.

Other interpretational issues should be also considered. For example, our projections are limited to the direct acute impact of heat and cold on mortality, and do not consider potentially larger impacts due to indirect pathways (e.g., sea-level rise or food production). Likewise, although the present study covers a large number of locations from different regions worldwide, it is not entirely representative of each geographic region or climate zone. For instance, temperature-related impacts in other warm regions such as Africa, the Arabian Peninsula, or other countries in South-East Asia such as India, were not explored due to the lack of observed temperature-mortality data. Indeed, these highly populated and mostly poor areas are particularly vulnerable to climate change, and it is expected that populations in these locations could suffer from more severe extreme heat events (Harrington et al. 2016; Russo et al. 2016; Lelieveld et al. 2016; Mazdiyasni et al. 2017).

We applied an analytical framework based on advanced, established statistical techniques that enabled us to model complex temperature-mortality associations and project the corresponding impacts under clearly defined warming scenarios (Gasparrini et al. 2017). Specifically, these methods allow modeling non-linear and lagged epidemiological relationships, capturing geographical differences, and separating the contributions of cold and heat temperatures (Gasparrini et al. 2015) (Supplementary Material S2). However, we also observe a large uncertainty in our estimates, particularly those for the net impacts and more extreme scenarios. The imprecision is attributed to variability in the climate models and to estimates of the exposure-response curves. In the latter, the uncertainty and potential biases generated in extrapolating the functions beyond the observed temperature range are not accounted for (Ebi and Rocklöv 2014; Benmarhnia et al. 2014). However, the impact of such extrapolation is unlikely to be substantial, because on average only 1.7 and 3.2% of heat days temperature were above the maximum observed in each 1.5- and 2-°C scenario (Table S7). Likewise, we should acknowledge that the results would likely be even less precise if other important sources of uncertainty, such as accounting for adaptation, were considered in the present analysis (Gosling et al. 2017).

## Conclusion

In conclusion, our findings suggest that, assuming no changes in population and vulnerability and consistent with the thresholds agreed in Paris, lower increases in GMT would reduce temperature-related mortality in most regions of the world. As reported in the 5th IPCC assessment report (IPCC 2014) and shown in the present study, the 2-°C long-term temperature goal should not be considered as “safe”, because warming at this level could still produce increments in mortality. However, conclusions on the additional benefits of an extra 0.5 °C decrease in GMT rise are not straightforward, with the comparison of 1.5 vs 2 °C affected by larger uncertainty and showing important geographical variability. Specifically, we found indications of a net increase in total mortality in cooler regions when restricting warming to 1.5 °C, due to the large component of cold-related mortality. In contrast, warmer areas could still experience a net decrease due to the relative rise in heat-related mortality. These patterns seem to follow a within-continent gradient according to the prevalent climate, with populations living in warmer areas, especially with equatorial climates, benefitting the most from stricter mitigation policies. Recent studies suggest that the direct consequences of climate change will be more visible in these tropical regions, not only in terms of increases in average temperature and a higher likelihood of more intense extreme-heat events, but also more frequent extreme weather episodes (i.e., droughts and floods) and more severe indirect effects (i.e., changes in crop yields) (Schleussner et al. 2016; Mora et al. 2017). In fact, these regions include the most populated areas worldwide and, in general, the most vulnerable to climate change, with a limited capacity for adaptation due to lack of infrastructural, financial, and technological resources (Smith et al. 2014).

## Electronic supplementary material

ESM 1 (DOCX 90 kb)

ESM 2 (PDF 84 kb)

ESM 3 (PDF 4 kb)

ESM 4 (PDF 85 kb)

ESM 5 (PDF 6 kb)
