# Supplementary material for: Temperature-related mortality impacts under and beyond Paris Agreement climate change scenarios
Source: Clim Change. 2018 Sep 13;150(3):391–402. doi: 10.1007/s10584-018-2274-3 (PMC6217994; doi:10.1007/s10584-018-2274-3)
Supplement: Supplementary file 1 — (DOCX 90 kb) [file 10584_2018_2274_MOESM1_ESM.docx]

**SUPPLEMENTARY MATERIAL - TABLES**

**Table S1.** Description of the 451 locations included in the analysis.

| **Gepgraphic Region** | **Country** | **N locations** | **Period** | **N deaths** |
| --- | --- | --- | --- | --- |
|  |  |  |  |  |
| *North America* | Canada | 26 | 1986 - 2011 | 2989901 |
|  | USA | 135 | 1985 - 2009 | 22953896 |
| *Central America* | Mexico | 10 | 1998 - 2014 | 2980086 |
| *South America* | Brazil | 18 | 1997 - 2011 | 3401136 |
|  | Chile | 4 | 2004 - 2014 | 325462 |
| *North Europe* | Finland | 1 | 1994 - 2011 | 130325 |
|  | Ireland | 6 | 1984 - 2007 | 1058215 |
|  | Sweden | 1 | 1990 - 2002 | 190092 |
|  | UK | 10 | 1990 - 2012 | 12075623 |
| *Central Europe* | Czech Republic | 4 | 1994 - 2015 | 711910 |
|  | France | 18 | 2000 - 2010 | 1197555 |
|  | Moldova | 4 | 2001 - 2010 | 59906 |
|  | Switzerland | 8 | 1995 - 2013 | 243638 |
| *South Europe* | Italy | 11 | 1987 - 2010 | 820390 |
|  | Spain | 52 | 1990 - 2014 | 3017110 |
| *East Asia* | China | 15 | 1996 - 2008 | 950130 |
|  | Japan | 47 | 1985 - 2012 | 26893197 |
|  | South Korea | 7 | 1992 - 2010 | 1726938 |
| *South-East Asia* | Philippines | 4 | 2006 - 2010 | 274516 |
|  | Taiwan | 3 | 1994 - 2007 | 765893 |
|  | Thailand | 62 | 1999 - 2008 | 1827853 |
|  | Vietnam | 2 | 2009 - 2013 | 108173 |
| *Australia* | Australia | 3 | 1988 - 2009 | 1177950 |

**Table S2.** GCM-specific 20-year periods with mean warming levels of 0.9°C, 1.4°C, 2.4°C, and 3.4°C above the 1986-2005 period, which correspond to 1.5°̧, 2°C, 3°C and 4°C above pre-industrial levels.

| **Warming level** | **Global Circulation Models** | | |
| --- | --- | --- | --- |
|  | **HadGEM2-ES** | **IPSL-CM5A-LR** | **MIROC-ESM-CHEM** |
| *1.5°C* | 2010 - 2029 | 2016 - 2035 | 2010 - 2029 |
| *2.0°C* | 2022 - 2041 | 2029 - 2048 | 2022 - 2041 |
| *3.0°C* | 2042 - 2061 | 2047 - 2066 | 2041 - 2060 |
| *4.0°C* | 2059 - 2078 | 2064 - 2083 | 2058 - 2077 |

**Table S3.** Excess mortality (%, 95% empirical confidence interval) due to cold, heat, and net (cold+heat) by country projected in each temperature-increase scenario.

| **Region** | **Country** | **1.5**°**C scenario** | | | **2**°**C scenario** | | | **3**°**C scenario** | | | **4**°**C scenario** | | |
| --- | --- | --- | --- | --- | --- | --- | --- | --- | --- | --- | --- | --- | --- |
|  |  | **Heat** | **Cold** | **Net** | **Heat** | **Cold** | **Net** | **Heat** | **Cold** | **Net** | **Heat** | **Cold** | **Net** |
| **North America** | *Canada* | 0.79  [0.24; 1.32] | 6.07  [3.25; 8.67] | 6.86  [3.8; 9.59] | 1.09  [0.35; 1.78] | 5.75  [3.04; 8.26] | 6.85  [3.86; 9.56] | 1.88  [0.62; 2.97] | 5.27  [2.75; 7.6] | 7.15  [4.16; 10.01] | 3.21  [1.05; 5.17] | 4.78  [2.50; 6.92] | 7.99  [4.55; 11.34] |
|  | *USA* | 0.63  [0.02; 1.19] | 4.89  [2.37; 7.23] | 5.51  [2.79; 7.96] | 0.91  [-0.01; 1.69] | 4.58  [2.19; 6.79] | 5.49  [2.76; 7.95] | 1.63  [-0.16; 3.16] | 4.12  [1.94; 6.16] | 5.75  [2.72; 8.64] | 2.55  [-0.36; 4.91] | 3.65  [1.72; 5.5] | 6.21  [2.39; 9.64] |
| **Central America** | *Mexico* | 0.61  [0.11; 1.12] | 4.18  [2.32; 6] | 4.79  [2.96; 6.56] | 1.16  [0.25; 1.99] | 3.45  [1.86; 5.06] | 4.61  [2.74; 6.36] | 2.29  [0.39; 4.14] | 2.53  [1.28; 3.75] | 4.82  [2.40; 7.08] | 4.14  [0.48; 7.85] | 1.79  [0.82; 2.72] | 5.93  [1.86; 9.84] |
| **South America** | *Brazil* | 1.43  [0.39; 2.43] | 2.12  [0.99; 3.29] | 3.55  [2.05; 4.86] | 2.13  [-0.16; 3.89] | 1.65  [0.76; 2.57] | 3.78  [1.24; 5.71] | 3.56  [-2.30; 7.49] | 1.20  [0.57; 1.88] | 4.77  [-1.21; 8.77] | 5.79  [-7.25; 13.7] | 0.78  [0.37; 1.25] | 6.56  [-6.68; 14.58] |
|  | *Chile* | 0.84  [0.24; 1.50] | 5.28  [2.25; 8.26] | 6.12  [3.00; 9.09] | 1.13  [0.33; 1.97] | 5.01  [2.09; 7.89] | 6.15  [2.98; 9.10] | 1.97  [0.53; 3.41] | 4.27  [1.63; 6.85] | 6.25  [2.98; 9.37] | 2.97  [0.74; 5.19] | 3.59  [1.18; 5.94] | 6.56  [2.88; 10.16] |
| **North Europe** | *Finland* | 0.92  [0.18; 1.66] | 6.87  [3.66; 9.78] | 7.80  [4.39; 10.87] | 1.23  [0.26; 2.13] | 6.45  [3.33; 9.42] | 7.68  [4.27; 10.82] | 1.75  [0.39; 3.00] | 5.58  [2.74; 8.34] | 7.33  [3.97; 10.49] | 2.99  [0.72; 4.88] | 4.80  [2.24; 7.38] | 7.80  [4.06; 11.38] |
|  | *Ireland* | 0.23  [-0.35; 0.73] | 7.90  [4.75; 10.65] | 8.13  [5.20; 10.76] | 0.34  [-0.50; 1.09] | 7.27  [4.34; 9.98] | 7.61  [4.86; 10.14] | 0.51  [-0.75; 1.53] | 6.64  [3.81; 9.18] | 7.14  [4.50; 9.58] | 0.89  [-1.28; 2.69] | 5.80  [3.23; 8.10] | 6.69  [3.96; 9.19] |
|  | *Sweden* | 0.60  [-0.29; 1.59] | 5.41  [2.09; 8.44] | 6.01  [2.48; 9.16] | 0.78  [-0.35; 1.97] | 5.03  [1.83; 8.07] | 5.81  [2.26; 9.00] | 1.13  [-0.50; 2.70] | 4.34  [1.47; 7.20] | 5.47  [1.96; 8.75] | 1.78  [-0.72; 3.95] | 3.74  [1.13; 6.41] | 5.52  [1.62; 9.20] |
|  | *UK* | 0.51  [0.20; 0.82] | 6.71  [4.72; 8.68] | 7.22  [5.13; 9.29] | 0.78  [0.31; 1.23] | 6.17  [4.26; 8.04] | 6.95  [4.90; 8.95] | 1.30  [0.55; 2.05] | 5.59  [3.71; 7.46] | 6.90  [4.67; 9.16] | 2.23  [1.00; 3.40] | 4.84  [3.12; 6.53] | 7.08  [4.68; 9.46] |
| **Central Europe** | *Czech Rep.* | 1.16  [0.56; 1.95] | 8.13  [5.14; 10.93] | 9.29  [6.14; 12.18] | 1.55  [0.82; 2.44] | 7.74  [4.82; 10.47] | 9.29  [6.18; 12.19] | 2.72  [1.33; 4.72] | 7.10  [4.39; 9.65] | 9.82  [6.45; 13.45] | 4.16  [2.31; 6.32] | 6.40  [3.86; 8.73] | 10.56  [7.13; 14.07] |
|  | *France* | 1.69  [1.02; 2.48] | 7.62  [4.67; 10.39] | 9.30  [6.19; 12.25] | 2.40  [1.53; 3.34] | 7.14  [4.30; 9.75] | 9.55  [6.47; 12.47] | 3.91  [2.50; 5.27] | 6.55  [3.84; 9.09] | 10.46  [7.21; 13.67] | 6.54  [4.75; 8.01] | 5.85  [3.39; 8.14] | 12.39  [9.04; 15.57] |
|  | *Moldova* | 1.76  [1.04; 2.43] | 10.85  [7.17; 14.25] | 12.61  [8.69; 15.94] | 2.26  [1.35; 3.09] | 10.42  [6.81; 13.74] | 12.68  [8.83; 16.1] | 3.89  [2.52; 5.17] | 9.63  [6.22; 12.74] | 13.52  [9.59; 17.15] | 6.17  [4.15; 7.82] | 8.74  [5.57; 11.63] | 14.90  [10.86; 18.77] |
|  | *Switzerland* | 1.16  [0.38; 2.06] | 5.19  [1.35; 8.68] | 6.35  [2.26; 9.96] | 1.67  [0.56; 2.87] | 4.89  [1.16; 8.26] | 6.55  [2.47; 10.22] | 2.86  [0.96; 5.13] | 4.41  [0.98; 7.55] | 7.26  [2.97; 11.47] | 4.76  [1.77; 7.68] | 3.92  [0.75; 6.8] | 8.68  [3.87; 13.26] |
| **South Europe** | *Italy* | 2.17  [1.55; 2.89] | 6.20  [3.29; 9.07] | 8.37  [5.35; 11.26] | 3.26  [2.39; 4.05] | 5.77  [2.94; 8.55] | 9.03  [5.92; 12.01] | 5.22  [3.78; 6.56] | 5.00  [2.33; 7.62] | 10.22  [6.85; 13.51] | 8.09  [6.09; 9.76] | 4.35  [1.90; 6.78] | 12.44  [8.89; 15.72] |
|  | *Spain* | 2.06  [1.29; 2.89] | 5.38  [2.32; 8.32] | 7.44  [4.29; 10.44] | 3.17  [2.00; 4.69] | 4.97  [2.10; 7.77] | 8.14  [4.88; 11.37] | 5.33  [3.45; 7.57] | 4.22  [1.57; 6.83] | 9.55  [6.02; 12.98] | 8.35  [6.07; 10.82] | 3.65  [1.23; 6.00] | 12.00  [8.33; 15.43] |
| **East Asia** | *China* | 1.16  [0.02; 2.36] | 10.33  [6.51; 13.74] | 11.49  [7.31; 15.12] | 1.63  [0.02; 3.15] | 9.66  [6.00; 12.97] | 11.29  [7.05; 14.99] | 2.71  [0.11; 5.05] | 8.49  [5.15; 11.58] | 11.20  [6.77; 15.22] | 4.29  [0.23; 7.4] | 7.57  [4.51; 10.30] | 11.86  [6.56; 16.55] |
|  | *Japan* | 0.46  [0.22; 0.74] | 7.94  [5.13; 10.49] | 8.40  [5.59; 11.06] | 0.67  [0.33; 1.06] | 7.37  [4.70; 9.83] | 8.04  [5.29; 10.62] | 1.17  [0.63; 1.70] | 6.58  [4.08; 8.88] | 7.74  [5.06; 10.33] | 1.84  [0.95; 2.76] | 5.81  [3.55; 7.93] | 7.66  [4.99; 10.16] |
|  | *South Korea* | 0.61  [0.18; 1.00] | 8.50  [5.43; 11.24] | 9.11  [6.06; 11.93] | 0.92  [0.29; 1.52] | 8.10  [5.14; 10.77] | 9.01  [6.08; 11.75] | 1.59  [0.54; 2.51] | 7.42  [4.73; 9.89] | 9.01  [6.15; 11.63] | 2.60  [0.89; 4.36] | 6.80  [4.31; 9.06] | 9.40  [6.38; 12.24] |
| **South-East Asia** | *Philippines* | 2.02  [1.27; 2.68] | 2.39  [0.94; 4.09] | 4.41  [2.74; 6.25] | 3.46  [1.79; 5.15] | 1.50  [0.51; 2.85] | 5.04  [3.16; 6.80] | 7.28  [2.78; 11.94] | 0.75  [0.19; 1.47] | 8.03  [3.57; 12.36] | 13.03  [3.35; 22.4] | 0.29  [0.05; 0.66] | 13.33  [3.67; 22.58] |
|  | *Taiwan* | 1.57  [0.56; 2.66] | 4.33  [2.67; 5.97] | 5.90  [4.14; 7.62] | 2.16  [0.76; 3.53] | 3.91  [2.38; 5.45] | 6.06  [4.13; 7.94] | 3.41  [0.94; 5.62] | 3.08  [1.73; 4.45] | 6.49  [3.67; 9.01] | 5.06  [0.96; 8.44] | 2.43  [1.26; 3.66] | 7.49  [3.11; 11.28] |
|  | *Thailand* | 2.87  [0.55; 4.85] | 2.05  [1.05; 3.06] | 4.91  [2.35; 7.08] | 4.36  [0.55; 7.31] | 1.40  [0.70; 2.13] | 5.76  [1.83; 8.82] | 7.15  [0.01; 11.98] | 0.84  [0.38; 1.34] | 7.98  [0.86; 12.83] | 11.82  [-2.14; 20.62] | 0.46  [0.16; 0.91] | 12.28  [-1.66; 21.19] |
|  | *Vietnam* | 2.06  [1.10; 2.98] | 2.70  [1.27; 4.21] | 4.76  [3.04; 6.44] | 4.19  [2.08; 6.72] | 1.72  [0.72; 2.81] | 5.91  [3.48; 8.70] | 8.58  [4.45; 11.85] | 0.75  [0.28; 1.29] | 9.33  [5.17; 12.73] | 16.88  [8.38; 23.19] | 0.31  [0.10; 0.59] | 17.19  [8.56; 23.54] |
| **Australia** | *Australia* | 0.64  [0.10; 1.21] | 7.27  [3.42; 10.63] | 7.91  [3.98; 11.49] | 0.84  [0.13; 1.60] | 6.60  [2.87; 9.94] | 7.44  [3.62; 10.95] | 1.38  [0.23; 2.75] | 5.56  [2.21; 8.60] | 6.94  [3.28; 10.45] | 2.18  [0.41; 4.23] | 4.52  [1.57; 7.23] | 6.70  [3.04; 10.36] |

**Table S4**. Excess mortality (%, 95% empirical confidence interval) due to cold, heat, and net (cold+heat) by geographic region and climate zone projected in each temperature-increase scenario.

|  | **1.5**°**C scenario** | | | **2**°**C scenario** | | | **3**°**C scenario** | | | **4**°**C scenario** | | |
| --- | --- | --- | --- | --- | --- | --- | --- | --- | --- | --- | --- | --- |
| **Geographic region** | **Heat** | **Cold** | **Net** | **Heat** | **Cold** | **Net** | **Heat** | **Cold** | **Net** | **Heat** | **Cold** | **Net** |
| *North America* | 0.64  [0.05; 1.2] | 5.01  [2.46; 7.38] | 5.66  [2.90; 8.13] | 0.93  [0.03; 1.69] | 4.71  [2.29; 6.94] | 5.64  [2.87; 8.12] | 1.66  [-0.08; 3.14] | 4.24  [2.02; 6.32] | 5.90  [2.86; 8.77] | 2.62  [-0.20; 4.92] | 3.77  [1.81; 5.65] | 6.40  [2.64; 9.80] |
| *Central America* | 0.61  [0.11; 1.12] | 4.18  [2.32; 6.00] | 4.79  [2.96; 6.56] | 1.16  [0.25; 1.99] | 3.45  [1.86; 5.06] | 4.61  [2.74; 6.36] | 2.29  [0.39; 4.14] | 2.53  [1.28; 3.75] | 4.82  [2.40; 7.08] | 4.14  [0.48; 7.85] | 1.79  [0.82; 2.72] | 5.93  [1.86; 9.84] |
| *South America* | 1.34  [0.38; 2.24] | 2.63  [1.20; 4.06] | 3.96  [2.25; 5.46] | 1.97  [-0.06; 3.55] | 2.19  [0.99; 3.41] | 4.15  [1.62; 6.12] | 3.31  [-1.74; 6.8] | 1.69  [0.75; 2.66] | 5.00  [-0.36; 8.71] | 5.34  [-5.97; 12.26] | 1.23  [0.51; 1.97] | 6.56  [-5.00; 13.70] |
| *North Europe* | 0.50  [0.15; 0.83] | 6.77  [4.71; 8.79] | 7.27  [5.11; 9.35] | 0.75  [0.23; 1.24] | 6.23  [4.25; 8.17] | 6.98  [4.87; 9.04] | 1.25  [0.44; 2.00] | 5.64  [3.69; 7.51] | 6.88  [4.65; 9.16] | 2.13  [0.79; 3.35] | 4.88  [3.13; 6.61] | 7.02  [4.59; 9.45] |
| *Central Europe* | 1.54  [0.9; 2.34] | 7.65  [4.57; 10.5] | 9.19  [5.95; 12.21] | 2.17  [1.32; 3.1] | 7.21  [4.25; 9.94] | 9.38  [6.20; 12.38] | 3.58  [2.15; 5.11] | 6.61  [3.82; 9.22] | 10.19  [6.80; 13.55] | 5.90  [4.03; 7.50] | 5.92  [3.36; 8.27] | 11.82  [8.33; 15.10] |
| *South Europe* | 2.09  [1.38; 2.77] | 5.63  [2.63; 8.55] | 7.73  [4.64; 10.67] | 3.20  [2.14; 4.42] | 5.22  [2.37; 8.01] | 8.41  [5.24; 11.52] | 5.30  [3.59; 7.09] | 4.46  [1.80; 7.06] | 9.76  [6.30; 13.00] | 8.27  [6.23; 10.17] | 3.87  [1.44; 6.21] | 12.13  [8.62; 15.43] |
| *East Asia* | 0.61  [0.21; 0.98] | 8.46  [5.47; 11.18] | 9.07  [6.05; 11.93] | 0.88  [0.30; 1.38] | 7.88  [5.01; 10.52] | 8.76  [5.77; 11.53] | 1.51  [0.55; 2.35] | 7.02  [4.39; 9.48] | 8.52  [5.59; 11.24] | 2.39  [0.89; 3.68] | 6.23  [3.83; 8.49] | 8.62  [5.55; 11.38] |
| *South-East Asia* | 2.43  [0.76; 3.88] | 2.55  [1.38; 3.75] | 4.98  [2.92; 6.79] | 3.81  [0.97; 6.05] | 1.89  [1.00; 2.8] | 5.70  [2.73; 8.2] | 6.65  [1.10; 10.57] | 1.20  [0.61; 1.81] | 7.85  [2.31; 11.9] | 11.29  [0.28; 18.44] | 0.76  [0.35; 1.23] | 12.05  [0.91; 19.31] |
| *Australia* | 0.64  [0.10; 1.21] | 7.27  [3.42; 10.63] | 7.91  [3.98; 11.49] | 0.84  [0.13; 1.60] | 6.60  [2.87; 9.94] | 7.44  [3.62; 10.95] | 1.38  [0.23; 2.75] | 5.56  [2.21; 8.60] | 6.94  [3.28; 10.45] | 2.18  [0.41; 4.23] | 4.52  [1.57; 7.23] | 6.70  [3.04; 10.36] |
| **Climate zone** |  |  |  |  |  |  |  |  |  |  |  |  |
| *A – Equatorial* | 2.08  [0.43; 3.52] | 1.82  [0.68; 2.98] | 3.90  [1.94; 5.61] | 3.30  [0.14; 5.77] | 1.26  [0.42; 2.12] | 4.56  [1.24; 7.18] | 5.76  [-1.20; 10.50] | 0.73  [0.18; 1.28] | 6.49  [-0.63; 11.35] | 9.65  [-4.96; 18.65] | 0.41  [0.05; 0.77] | 10.06  [-4.70; 19.12] |
| *B – Arid* | 0.83  [0.21; 1.43] | 4.14  [0.92; 7.21] | 4.98  [1.68; 8.04] | 1.37  [0.37; 2.29] | 3.77  [0.73; 6.66] | 5.14  [1.86; 8.17] | 2.35  [0.62; 3.94] | 3.22  [0.50; 5.78] | 5.57  [2.09; 8.88] | 3.80  [0.86; 6.51] | 2.72  [0.29; 4.98] | 6.52  [2.36; 10.47] |
| *C – Warm temperate* | 0.74  [0.32; 1.13] | 6.51  [4.06; 8.87] | 7.25  [4.73; 9.62] | 1.07  [0.47; 1.62] | 6.01  [3.69; 8.23] | 7.08  [4.60; 9.38] | 1.84  [0.74; 2.79] | 5.30  [3.17; 7.35] | 7.14  [4.62; 9.53] | 2.98  [1.12; 4.45] | 4.61  [2.63; 6.44] | 7.59  [4.75; 10.23] |
| *D – Snow* | 0.78  [0.18; 1.35] | 7.59  [4.64; 10.34] | 8.37  [5.30; 11.21] | 1.15  [0.27; 1.89] | 7.17  [4.38; 9.8] | 8.32  [5.20; 11.15] | 1.96  [0.51; 3.18] | 6.57  [3.95; 9.02] | 8.53  [5.35; 11.38] | 3.16  [0.89; 5.14] | 5.97  [3.55; 8.15] | 9.13  [5.61; 12.46] |

**Table S5.** Difference in excess mortality (%, 95% empirical confidence interval) due to cold, heat, and net (cold+heat) by country projected in each temperature-increase scenario, relative to 1.5°C.

| **Region** | **Country** | **2**°**C vs 1.5**°**C** | | | **3**°**C vs 1.5**°**C** | | | **4**°**C vs 1.5**°**C** | | |
| --- | --- | --- | --- | --- | --- | --- | --- | --- | --- | --- |
|  |  | **Heat** | **Cold** | **Net** | **Heat** | **Cold** | **Net** | **Heat** | **Cold** | **Net** |
| **North America** | *Canada* | +0.30  [+0.10; +0.54] | -0.31  [-0.53; -0.14] | -0.01  [-0.31; +0.26] | +1.09  [+0.37; +1.69] | -0.80  [-1.25; -0.40] | +0.29  [-0.49; +0.95] | +2.42  [+0.83; +3.87] | -1.29  [-1.94; -0.67] | +1.13  [-0.51; +2.56] |
|  | *USA* | +0.28  [-0.03; +0.55] | -0.31  [-0.49; -0.15] | -0.02  [-0.37; +0.25] | +1.01  [-0.18; +1.97] | -0.77  [-1.14; -0.41] | +0.24  [-0.92; +1.18] | +1.93  [-0.39; +3.71] | -1.24  [-1.79; -0.66] | +0.69  [-1.55; +2.40] |
| **Central America** | *Mexico* | +0.54  [+0.10; +0.92] | -0.73  [-1.25; -0.26] | -0.18  [-0.88; +0.44] | +1.68  [+0.22; +3.13] | -1.65  [-2.36; -0.97] | +0.03  [-1.54; +1.47] | +3.53  [+0.30; +6.83] | -2.39  [-3.32; -1.47] | +1.14  [-2.24; +4.41] |
| **South America** | *Brazil* | +0.70  [-0.68; +1.61] | -0.47  [-0.86; -0.19] | +0.23  [-1.10; +1.24] | +2.13  [-2.84; +5.57] | -0.92  [-1.45; -0.40] | +1.21  [-3.62; +4.65] | +4.36  [-8.01; +11.72] | -1.34  [-2.11; -0.60] | +3.01  [-8.98; +10.29] |
|  | *Chile* | +0.29  [+0.07; +0.49] | -0.27  [-0.43; -0.11] | +0.03  [-0.23; +0.28] | +1.14  [+0.27; +1.93] | -1.01  [-1.53; -0.51] | 0.13  [-0.84; +1.15] | +2.13  [+0.45; +3.75] | -1.69  [-2.49; -0.89] | +0.44  [-1.48; +2.32] |
| **North Europe** | *Finland* | +0.30  [+0.07; +0.50] | -0.42  [-0.86; +0.00] | -0.12  [-0.61; +0.33] | +0.82  [+0.21; +1.36] | -1.29  [-2.13; -0.67] | -0.46  [-1.43; +0.27] | +2.07  [+0.57; +3.36] | -2.07  [-3.33; -1.12] | +0.00  [-2.01; +1.79] |
|  | *Ireland* | +0.11  [-0.16; +0.36] | -0.63  [-1.04; -0.25] | -0.51  [-1.04; -0.08] | +0.28  [-0.41; +0.83] | -1.26  [-1.64; -0.89] | -0.98  [-1.85; -0.28] | +0.66  [-0.96; +1.98] | -2.09  [-2.63; -1.52] | -1.43  [-3.29; +0.11] |
|  | *Sweden* | +0.17  [-0.06; +0.40] | -0.38  [-0.79; -0.05] | -0.20  [-0.67; +0.13] | +0.53  [-0.21; +1.15] | -1.07  [-1.89; -0.48] | -0.54  [-1.60; +0.30] | +1.18  [-0.42; +2.46] | -1.67  [-2.83; -0.77] | -0.49  [-2.41; +0.99] |
|  | *UK* | +0.27  [+0.11; +0.42] | -0.54  [-0.86; -0.26] | -0.27  [-0.61; +0.01] | +0.79  [+0.34; +1.27] | -1.12  [-1.45; -0.78] | -0.33  [-0.95; +0.34] | +1.72  [+0.80; +2.60] | -1.87  [-2.30; -1.46] | -0.15  [-1.17; +0.83] |
| **Central Europe** | *Czech Rep.* | +0.39  [+0.22; +0.55] | -0.38  [-0.73; -0.20] | +0.00  [-0.44; +0.30] | +1.56  [+0.72; +2.79] | -1.03  [-1.52; -0.55] | +0.53  [-0.58; +2.07] | +3.00  [+1.75; +4.39] | -1.73  [-2.43; -1.12] | +1.27  [-0.21; +2.90] |
|  | *France* | +0.72  [+0.49; +0.92] | -0.47  [-0.77; -0.24] | +0.25  [-0.15; +0.60] | +2.22  [+1.45; +2.88] | -1.07  [-1.54; -0.60] | +1.15  [+0.15; +2.00] | +4.85  [+3.55; + 5.95] | -1.77  [-2.49; -1.13] | +3.09  [+1.72; +4.26] |
|  | *Moldova* | +0.50  [+0.31; +0.69] | -0.42  [-0.78; -0.16] | +0.08  [-0.25; +0.40] | +2.14  [+1.23; +3.32] | -1.22  [-1.85; -0.53] | +0.91  [-0.32; +1.91] | +4.41  [+2.90; +5.79] | -2.11  [-2.93; -1.36] | +2.30  [+0.86; +3.72] |
|  | *Switzerland* | +0.51  [+0.18+; +0.81] | -0.30  [-0.64; -0.10] | +0.21  [-0.27; +0.60] | +1.70  [+0.58; +3.08] | -0.78  [-1.41; -0.25] | +0.91  [-0.48; +2.57] | +3.60  [+1.41; +5.65] | -1.26  [-2.15; -0.47] | +2.34  [+0.03; +4.57] |
| **South Europe** | *Italy* | +1.09  [+0.80; +1.39] | -0.43  [-0.75; -0.21] | +0.66  [+0.24; +1.03] | +3.05  [+2.25; +3.72] | -1.19  [-1.85; -0.56] | +1.85  [+0.71; +2.89] | +5.91  [+4.50; +7.05] | -1.84  [-2.67; -1.12] | +4.07  [+2.65; +5.27] |
|  | *Spain* | +1.11  [+0.67; +1.80] | -0.41  [-0.66; -0.21] | +0.70  [+0.19; +1.44] | +3.27  [+2.15; +4.70] | -1.16  [-1.69; -0.60] | +2.11  [+0.81; +3.51] | +6.29  [+4.61; +7.94] | -1.73  [-2.46; -0.98] | +4.56  [+2.92; +6.25] |
| **East Asia** | *China* | +0.47  [-0.01; +0.83] | -0.67  [-0.95; -0.38] | -0.20  [-0.72; +0.21] | +1.55  [+0.09; +2.70] | -1.84  [-2.54; -1.23] | -0.29  [-1.84; +0.89] | +3.13  [+0.19; +5.41] | -2.76  [-3.52; -1.89] | +0.37  [-2.63; +2.69] |
|  | *Japan* | +0.20  [+0.06; +0.42] | -0.57  [-1.15; -0.08] | -0.36  [-0.84; +0.01] | +0.70  [+0.36; +1.07] | -1.37  [-1.86; -0.82] | -0.66  [-1.18; -0.18] | +1.38  [+0.73; +2.07] | -2.13  [-2.94; -1.46] | -0.75  [-1.57; +0.04] |
|  | *South Korea* | +0.31  [+0.11; +0.60] | -0.41  [-0.78; -0.13] | -0.10  [-0.45; +0.18] | +0.98  [+0.36; +1.51] | -1.08  [-1.57; -0.65] | -0.10  [-0.87; +0.62] | +1.99  [+0.71; +3.37] | -1.71  [-2.33; -1.06] | +0.29  [-1.15; +1.84] |
| **South-East Asia** | *Philippines* | +1.44  [+0.50; +2.60] | -0.81  [-1.25; -0.38] | +0.63  [-0.46; +1.94] | +5.26  [+1.44; +9.44] | -1.64  [-2.71; -0.72] | +3.62  [-0.50; +8.20] | +11.01  [+1.98; +19.99] | -2.09  [-3.53; -0.88] | +8.92  [-0.16; +18.49] |
|  | *Taiwan* | +0.58  [+0.10; +1.05] | -0.42  [-0.69; -0.19] | +0.16  [-0.38; +0.59] | +1.84  [+0.13; +3.34] | -1.25  [-1.72; -0.84] | +0.59  [-1.16; +2.10] | +3.48  [-0.11; +6.43] | -1.90  [-2.51; -1.30] | +1.59  [-2.04; +4.55] |
|  | *Thailand* | +1.49  [-0.10; +2.63] | -0.64  [-1.09; -0.32] | +0.85  [-0.79; +1.93] | +4.28  [-0.67; +7.53] | -1.21  [-1.80; -0.65] | +3.07  [-1.87; +6.36] | +8.95  [-2.97; +16.45] | -1.59  [-2.43; -0.83] | +7.36  [-4.34; +14.80] |
|  | *Vietnam* | +2.13  [+0.93; +3.97] | -0.98  [-1.46; -0.52] | +1.15  [-0.15; +2.98] | +6.52  [+3.29; +9.05] | -1.95  [-2.96; -0.97] | +4.57  [+1.18; +7.22] | +14.82  [+6.97; +20.46] | -2.39  [-3.73; -1.14] | +12.43  [+4.65; +17.98] |
| **Australia** | *Australia* | +0.20  [+0.03; +0.39] | -0.67  [-0.96; -0.39] | -0.47  [-0.76; -0.18] | +0.73  [+0.13; +1.58] | -1.71  [-2.32; -1.06] | -0.97  [-1.75; -0.01] | +1.54  [+0.29; +3.09] | -2.74  [-3.77; -1.67] | -1.21  [-2.69; +0.54] |

**Table S6.** Difference in excess mortality (%, 95% empirical confidence interval) due to cold, heat, and net (cold+heat) by geographic region and climate zone in each temperature-increase scenario, relative to 1.5°C.

|  | **2**°**C vs 1.5**°**C** | | | **3**°**C vs 1.5**°**C** | | | **4**°**C vs 1.5**°**C** | | |
| --- | --- | --- | --- | --- | --- | --- | --- | --- | --- |
| **Geographic region** | **Heat** | **Cold** | **Net** | **Heat** | **Cold** | **Net** | **Heat** | **Cold** | **Net** |
| *North America* | +0.29  [-0.02; +0.55] | -0.31  [-0.49; -0.15] | -0.02  [-0.35; +0.25] | +1.02  [-0.12; +1.94] | -0.78  [-1.15; -0.41] | +0.24  [-0.87; +1.14] | +1.98  [-0.25; +3.72] | -1.24  [-1.80; -0.66] | +0.74  [-1.42; +2.41] |
| *Central America* | +0.54  [+0.10; +0.92] | -0.73  [-1.25; -0.26] | -0.18  [-0.88; +0.44] | +1.68  [+0.22; +3.13] | -1.65  [-2.36; -0.97] | +0.03  [-1.54; +1.47] | +3.53  [+0.30; +6.83] | -2.39  [-3.32; -1.47] | +1.14  [-2.24; +4.41] |
| *South America* | +0.63  [-0.56; +1.43] | -0.44  [-0.78; -0.19] | +0.19  [-0.97; +1.08] | +1.97  [-2.34; +4.97] | -0.93  [-1.43; -0.44] | +1.04  [-3.19; +4.03] | +4.00  [-6.56; +10.44] | -1.40  [-2.15; -0.66] | +2.60  [-7.80; +8.96] |
| *North Europe* | +0.25  [+0.09; +0.41] | -0.54  [-0.87; -0.26] | -0.29  [-0.64; +0.00] | +0.75  [+0.27; +1.22] | -1.13  [-1.47; -0.78] | -0.38  [-1.03; +0.28] | +1.63  [+0.64; +2.55] | -1.88  [-2.34; -1.45] | -0.25  [-1.34; +0.77] |
| *Central Europe* | +0.63  [+0.41; +0.82] | -0.44  [-0.75; -0.23] | +0.19  [-0.22; +0.51] | +2.04  [+1.24; +2.80] | -1.04  [-1.53; -0.56] | +1.00  [-0.05; +2.03] | +4.36  [+3.03; +5.41] | -1.73  [-2.46; -1.09] | +2.63  [+1.22; +3.78] |
| *South Europe* | +1.10  [+0.71; +1.68] | -0.41  [-0.69; -0.21] | +0.69  [+0.21; +1.31] | +3.20  [+2.19; +4.35] | -1.17  [-1.72; -0.59] | +2.03  [+0.78; +3.15] | +6.17  [+4.66; +7.48] | -1.76  [-2.49; -1.03] | +4.41  [+2.92; +5.75] |
| *East Asia* | +0.27  [+0.07; +0.49] | -0.58  [-1.08; -0.15] | -0.31  [-0.78; +0.06] | +0.89  [+0.34; +1.40] | -1.44  [-1.94; -0.93] | -0.55  [-1.24; +0.07] | +1.77  [+0.66; +2.74] | -2.23  [-2.96; -1.54] | -0.45  [-1.69; +0.68] |
| *South-East Asia* | +1.38  [+0.15; +2.23] | -0.66  [-0.98; -0.36] | +0.72  [-0.55; +1.62] | +4.22  [+0.17; +7.08] | -1.35  [-1.98; -0.75] | +2.87  [-1.27; +5.77] | +8.86  [-0.8; +15.17] | -1.79  [-2.69; -0.98] | +7.07  [-2.45; +13.46] |
| *Australia* | +0.20  [+0.03; +0.39] | -0.67  [-0.96; -0.39] | -0.47  [-0.76; -0.18] | +0.73  [+0.13; +1.58] | -1.71  [-2.32; -1.06] | -0.97  [-1.75; -0.01] | +1.54  [+0.29; +3.09] | -2.74  [-3.77; -1.67] | -1.21  [-2.69; +0.54] |
| **Climate zone** |  |  |  |  |  |  |  |  |  |
| *A – Equatorial* | +1.22  [-0.34; +2.28] | -0.55  [-0.88; -0.24] | +0.66  [-0.91; +1.81] | 3.68  [-1.85; 7.47] | -1.09  [-1.72; -0.47] | +2.59  [-2.86; +6.36] | +7.57  [-5.80; +15.58] | -1.41  [-2.29; -0.58] | +6.16  [-6.93; +14.27] |
| *B – Arid* | +0.53  [+0.13; +0.87] | -0.37  [-0.59; -0.14] | +0.16  [-0.29; +0.55] | 1.52  [0.34; 2.55] | -0.93  [-1.45; -0.36] | +0.59  [-0.64; +1.67] | +2.97  [0.58; +5.08] | -1.43  [-2.27; -0.53] | +1.54  [-0.89; +3.70] |
| *C – Warm temperate* | +0.33  [+0.12; +0.53] | -0.50  [-0.84; -0.27] | -0.17  [-0.53; +0.08] | 1.11  [0.40; 1.69] | -1.21  [-1.61; -0.81] | -0.10  [-0.87; 0.57] | +2.24  [0.77; +3.37] | -1.90  [-2.47; -1.33] | +0.34  [-1.14; +1.49] |
| *D – Snow* | +0.36  [+0.09; +0.60] | -0.42  [-0.66; -0.18] | -0.05  [-0.39; +0.26] | 1.18  [0.33; 1.89] | -1.02  [-1.44; -0.65] | +0.16  [-0.71; +0.87] | +2.38  [0.70; +3.78] | -1.62  [-2.17; -1.02] | +0.76  [-0.93; +2.18] |

**Table S7.** Ratio between number of days and corresponding heat-related deaths with mean temperature above the maximum registered in the observed period and the total number of days and deaths with mean temperature above MMT, projected under scenarios 1.5°C and 2°C.

| **Geographic Region** | **Country** | **Ratio N days _T>max_ / N days _T>MMT_** | | **Ratio N deaths _T>max_ / N deaths _T>MMT_** | |
| --- | --- | --- | --- | --- | --- |
|  |  | **1.5°C scenario** | **2°C scenario** | **1.5°C scenario** | **2°C scenario** |
| **Overall** |  | 0.017 | 0.032 | 0.10 | 0.14 |
| *North America* | Canada | 0.005 | 0.010 | 0.01 | 0.03 |
|  | USA | 0.009 | 0.017 | 0.06 | 0.09 |
| *Central America* | Mexico | 0.006 | 0.019 | 0.04 | 0.09 |
| *South America* | Brazil | 0.012 | 0.035 | 0.09 | 0.14 |
|  | Chile | 0.020 | 0.028 | 0.04 | 0.04 |
| *North Europe* | Finland | 0.079 | 0.108 | 0.06 | 0.10 |
|  | Ireland | 0.888 | 0.899 | 0.09 | 0.11 |
|  | Sweden | 0.012 | 0.038 | 0.01 | 0.05 |
|  | UK | 0.041 | 0.046 | 0.02 | 0.03 |
| *Central Europe* | Czech Rep. | 0.013 | 0.029 | 0.03 | 0.07 |
|  | France | 0.004 | 0.007 | 0.01 | 0.01 |
|  | Moldova | 0.005 | 0.010 | 0.02 | 0.04 |
|  | Switzerland | 0.015 | 0.030 | 0.03 | 0.06 |
| *South Europe* | Italy | 0.006 | 0.016 | 0.05 | 0.09 |
|  | Spain | 0.007 | 0.015 | 0.04 | 0.07 |
| *East Asia* | China | 0.026 | 0.041 | 0.24 | 0.30 |
|  | Japan | 0.006 | 0.017 | 0.05 | 0.11 |
|  | South Korea | 0.007 | 0.016 | 0.02 | 0.05 |
| *South-East Asia* | Philippines | 0.006 | 0.018 | 0.10 | 0.15 |
|  | Taiwan | 0.007 | 0.014 | 0.06 | 0.10 |
|  | Thailand | 0.032 | 0.057 | 0.28 | 0.34 |
|  | Vietnam | 0.008 | 0.022 | 0.17 | 0.28 |
| *Australia* | Australia | 0.001 | 0.001 | 0.01 | 0.01 |

|  |  | **HadGEM2-ES** | | | | **IPSL-CM5A-LR** | | | | **MIROC-ESM-CHEM** | | | |
| --- | --- | --- | --- | --- | --- | --- | --- | --- | --- | --- | --- | --- | --- |
|  |  | 1.5°C scenario | | 2°C scenario | | 1.5°C scenario | | 2°C scenario | | 1.5°C scenario | | 2°C scenario | |
|  |  | Cold | Heat | Cold | Heat | Cold | Heat | Cold | Heat | Cold | Heat | Cold | Heat |
| *North America* | Canada | -0.7 | -15.1 | -2.1 | -8.2 | -0.8 | 4.8 | -0.8 | -3.6 | 1.5 | 10.4 | 2.9 | 11.8 |
|  | USA | -0.8 | -14.5 | -2.5 | -6.1 | -1.9 | 11.5 | -1.4 | 3.1 | 2.8 | 3.0 | 4.0 | 2.9 |
| *Central America* | Mexico | -2.4 | 15.7 | -2.2 | 5.3 | -1.7 | -12.9 | 6.4 | -3.1 | 4.0 | -2.8 | -4.2 | -2.2 |
| *South America* | Brazil | -7.7 | 8.8 | -0.1 | 6.7 | 2.4 | 3.7 | -0.2 | -0.2 | 5.4 | -12.5 | 0.2 | -6.5 |
|  | Chile | -4.5 | 18.3 | -4.0 | 16.2 | 3.4 | -22.8 | 1.8 | -17.5 | 1.1 | 4.5 | 2.2 | 1.3 |
| *North Europe* | Finland | -1.4 | 3.3 | -6.9 | 4.2 | 0.7 | -18.3 | 4.9 | -13.8 | 0.7 | 15.0 | 2.0 | 9.6 |
|  | Ireland | -2.7 | -7.1 | -2.0 | -5.6 | 1.0 | -10.1 | 4.6 | -15.4 | 1.7 | 17.2 | -2.5 | 21.0 |
|  | Sweden | -4.2 | 28.8 | -10.0 | 29.2 | 2.2 | -38.0 | 6.7 | -34.0 | 2.1 | 9.2 | 3.3 | 4.8 |
|  | UK | -4.4 | -11.4 | -4.5 | -10.6 | 0.2 | -3.8 | 3.8 | -5.5 | 4.2 | 15.2 | 0.7 | 16.1 |
| *Central Europe* | Czech Republic | 1.0 | -32.7 | 0.0 | -25.4 | 0.0 | 9.7 | 2.6 | 11.7 | -1.0 | 23.0 | -2.7 | 13.7 |
|  | France | -0.5 | -25.8 | -0.6 | -18.7 | -1.6 | 20.9 | 0.4 | 17.2 | 2.1 | 4.9 | 0.1 | 1.5 |
|  | Moldova | 2.0 | -9.9 | 0.0 | -8.1 | 0.1 | -7.3 | 2.3 | -9.0 | -2.0 | 17.2 | -2.3 | 17.1 |
|  | Switzerland | 2.4 | -27.7 | 1.0 | -25.3 | -2.1 | 15.4 | 0.9 | 15.7 | -0.3 | 12.4 | -1.9 | 9.6 |
| *South Europe* | Italy | 2.7 | -15.1 | 0.4 | -7.1 | -1.8 | 9.6 | 1.9 | 7.3 | -0.9 | 5.4 | -2.2 | -0.2 |
|  | Spain | 2.7 | -2.2 | 1.4 | 6.8 | -5.4 | 23.0 | -3.7 | 21.5 | 2.7 | -20.7 | 2.3 | -28.4 |
| *East Asia* | China | -2.2 | 3.4 | -4.3 | 5.1 | 1.0 | 27.7 | 2.3 | 19.4 | 1.2 | -31.1 | 1.9 | -24.5 |
|  | Japan | 1.5 | -16.2 | -4.4 | 4.7 | -1.8 | -4.4 | 3.6 | -20.6 | 0.3 | 20.6 | 0.8 | 15.9 |
|  | South Korea | 0.2 | -5.4 | -2.8 | 3.6 | -0.5 | 2.4 | 2.1 | -10.5 | 0.3 | 3.0 | 0.7 | 6.9 |
| *South-East Asia* | Philippines | -12.5 | 7.6 | -19.2 | 13.8 | 29.1 | -3.5 | 38.2 | -16.7 | -16.6 | -4.2 | -19.0 | 2.9 |
|  | Taiwan | 0.4 | 2.2 | -3.8 | 5.6 | -2.3 | 4.5 | 1.1 | -1.4 | 1.9 | -6.8 | 2.7 | -4.2 |
|  | Thailand | -8.8 | 8.1 | 1.5 | 1.4 | 0.0 | 6.7 | -8.2 | 9.1 | 8.8 | -14.8 | 6.6 | -10.5 |
|  | Vietnam | 12.4 | 3.5 | 16.3 | 15.1 | -5.0 | 1.1 | -9.4 | 9.4 | -7.4 | -4.6 | -6.9 | -24.5 |
| *Australia* | Australia | 0.5 | -6.5 | 0.7 | -8.5 | 1.2 | -16.3 | 2.7 | -18.9 | -1.7 | 22.8 | -3.3 | 27.4 |

**Table S8**. Coefficients of variation of the temperature-mortality estimates obtained from each of the Global Climatic Models (GCMs). Results correspond to the percentage of the difference in attributable number of deaths between the ensemble model and the corresponding GCM, and the attributable number in the ensemble model in each country for heat and cold.

**SUPPLEMENTARY MATERIAL - FIGURES**

**Figure S1.** Map showing the geographical distribution of the locations included in the study classified into 4 big climate zones, defined according to the Köppen-Geiger classification^15^.

**Figure S2.** Map showing the location-specific increase in modelled mean temperature (°C, GCM-ensemble) from 1.5°C to 2°C scenario.

**Figure S3.** Temporal trends in increase in modelled mean temperature (°C, GCM-ensemble) from pre-industrial levels averaged across the included locations in the study.

**Figure S4**. Difference in GCM-specific and ensemble mortality impacts between 1.5°C and 2°C scenario across geographic regions.
