# Supplementary figures and images for: Temperature-related mortality impacts under and beyond Paris Agreement climate change scenarios
Source: Clim Change. 2018 Sep 13;150(3):391–402. doi: 10.1007/s10584-018-2274-3 (PMC6217994; doi:10.1007/s10584-018-2274-3)

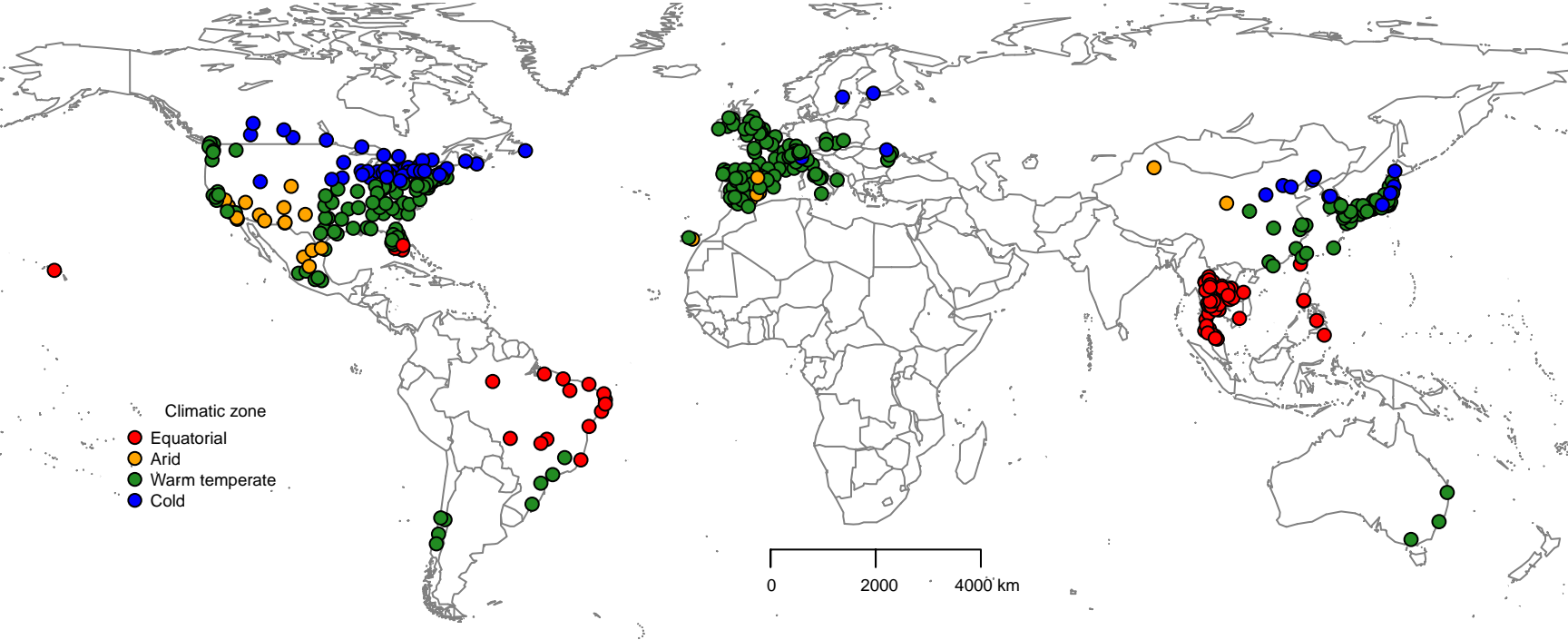

Supplement: Supplementary file 2 — (PDF 84 kb) [file 10584_2018_2274_MOESM2_ESM.pdf]

Increase in temperature (°C)

6.0  
5.5  
5.0  
4.5  
4.0  
3.5  
3.0  
2.5  
2.0  
1.5  
1.0  
0.5  
0.0

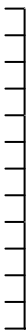

2000-09

2020-29

2040-49

2060-69

2080-89

Year

Supplement: Supplementary file 3 — (PDF 4 kb) [file 10584_2018_2274_MOESM3_ESM.pdf]

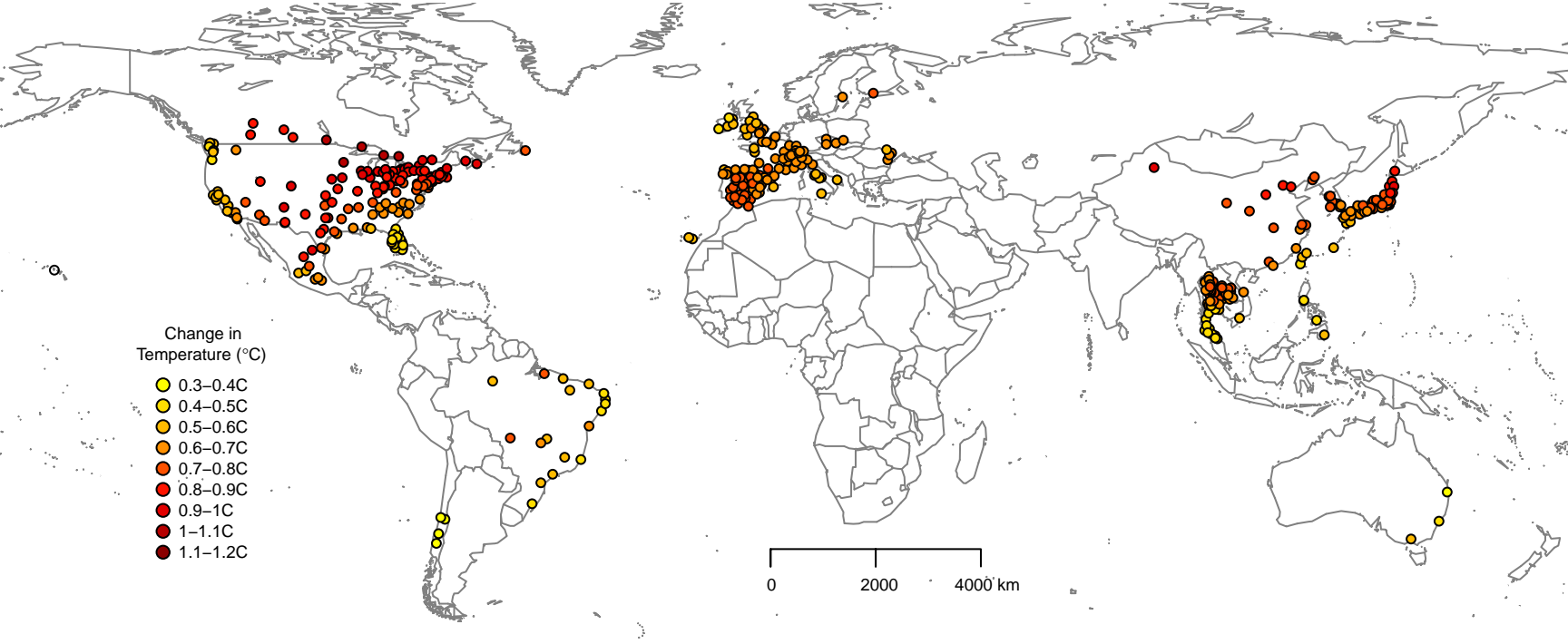

Supplement: Supplementary file 4 — (PDF 85 kb) [file 10584_2018_2274_MOESM4_ESM.pdf]

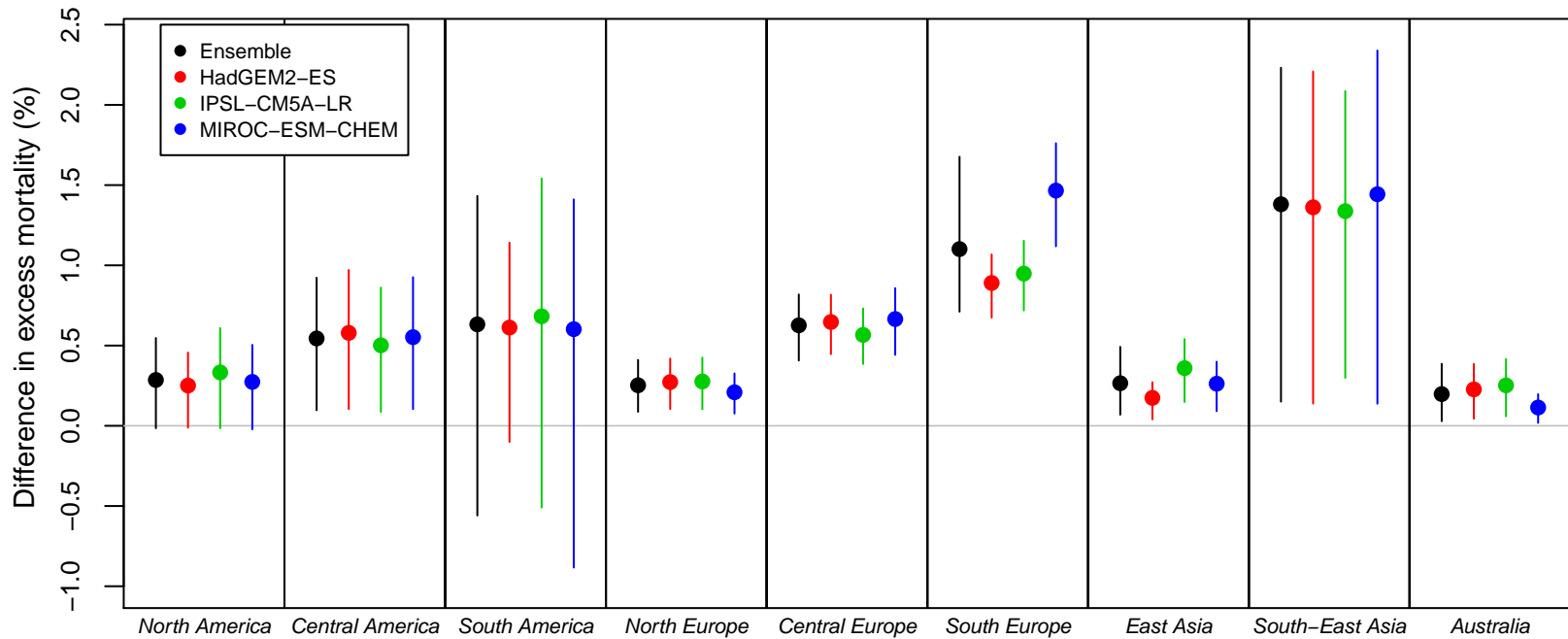

Supplement: Supplementary file 5 — (PDF 6 kb) [file 10584_2018_2274_MOESM5_ESM.pdf]
